# The Austin Flood

**Published:** 1911-11

**Authors:** 


### The Austin Flood

THE Boro of Austin had, at the time of the flood, about 3,000 population. It is situated in a narrow valley at the junction of a small stream flowing east from Keating Summit, and Freeman Run, a very rapid stream flowing southward from Odin, the latter being the main stream which was used by the Bayless Paper Mill.

The industries of Austin consisted of a hard wood mill at the lower end of town, a kindling wood factory and hemlock mill, both just below Main Street, and the Bayless Paper Mill situated about one-quarter mile above the town and in the valley of Freeman Run.

The hospital and school buildings being on a promontory, were not affected by the flood.

The town was practically heated and lighted by natural gas.

The Bayless Paper Company had built a large concrete dam across Freeman Run about two miles above Austin, to regulate the flow of water to the paper mill.

Main Street ran directly across Freeman Run, and between Main Street, and the paper mill there is a flat about 100 rods wide and this flat was the principal residence part of the town.

Above the paper mill, toward the dam, were stored several thousand cords of 4-foot pulp wood.

When the dam broke, three sections, each 30 to 40 feet long, moved downward, opening a gateway at least 100 feet wide, allowing the one-half mile of artificial lake a sudden outlet down the valley.

The thousands of cords of pulp wood were first moved by the water, this mass lodged against the buildings below, until they were pushed from their foundations, and these buildings in turn lodged against other buildings, the force and mass of buildings increasing until the whole residence portion of the flat was swept clean of all buildings to Main Street, both sides of which were built mainly of brick. Some wooden buildings on each end of Main Street finally gave way allowing two channels to form, one into the mill pond and against the hemlock mill, and the other down the course of the main stream, through Costello 3 miles below, and gradually fading out down the Sinnemahoning.

The break in the dam was discovered by a resident on the nearby hillside who fortunately had a telephone and promptly telephoned to Austin, two miles away. "The dam has broken." The Central promptly turned in a fire alarm, the only thing she could do, then fled to the street crying "The dam has broken." The news spread rapidly, and this saved the lives of hundreds of people.

Very soon the Central telephone station was destroyed and all outside communication by 'phone was cut off.

A man ran from the dam to Odin 4 miles above and telephoned the news to the surrounding towns.

This caused considerable delay, but the news reached Coudersport about 4 P. M.

I arrived on the scene about 5 P. M. and being the County Medical Inspector for the Pennsylvania Department of Health, and the first out of town physician on the ground, I felt it my duty to take charge of the hospital and have remained in charge since, especially as all of the local physicians resided in the flooded district and were occupied with their own troubles.

A careful census made by two competent persons continued over a period of several days visiting individuals representing every family in town, places the loss of life at 75, which does not include outsiders who may have been in town at the time.

At this date, 62 bodies have been recovered. Soon after the flood, fire broke out in three different places, gas lines were broken which fed the flames.

The dam supplied water for fire purposes, but this was gone.

Fire engines from Renova and Smethport arrived about 11 o'clock which saved a general conflagration.

During the week following 9 flood victims have been admitted to the hospital, representing all of the injured and seriously sick as the result of the flood.

All of those admitted were surgical cases as follows.

2 fractured clavicles.

1 fractured thigh (lower third).

1 fractured leg (upper third).

1 rupture of internal lateral ligaments of knee.

1 punctured wound of foot, infected, developed abscess.

All of the above as well as the remaining ones suffered from numerous contusions, principally about the head.

One man with fractured clavicle developed pneumonia and died, all the others are recovering nicely; in fact, all but the one with the broken legs are up and around.

Judging from these and the bodies I have seen in the morgue, I am convinced that the majority died from violence by falling timbers, and being caught in buildings that were crushed.

All of the five physicians lost all their instruments and libraries. Dr. E. A. Mansuy lost his wife.

E. H. Ashcraft,
Coudersport, Pa.

---

Dr. Roy J. Juhre, of Hinsdale, a former student of the editor, and a Red Cross "Minute Man," was preparing to go to Austin on the first train of the day following the flood, when Mr. Almy and others of the Buffalo Charity Organization Society called for him in an automobile and he went breakfastless with them. Some of his observations bring out new points, which we have not seen in the press dispatches. One of the most important is that the flood was anticipated. Material for the reconstruction of the dam was already on the ground. The Pulp Mill management had a chain and ring prepared and a man on duty at the dam to fasten the chain so as to blow a continuous blast of the whistle as a flood warning. This form of signal was arranged with the idea that it could be put into action instantly and allow time for the man on duty to escape to the high ground. Unfortunately, the man on duty at the time that the dam gave way had more heroism than forethought and he stayed at his post and pulled the chain thirteen times, thus giving the fire signal and calling the people toward the danger point. The owner of the property next to Senator Baldwin's had cut an opening in a barb-wire fence to afford quick access to the hill and it was probably due to the fact that Mr. Baldwin was blind and his wife crippled that they missed this opening in their attempt at escape. The press story regarding the delay of the telephonic warning was also denied.

Owing to the confusion of flood and fire signals, loss of life in the low-lying business section became inevitable. The brick buildings of this part of the town acted as a secondary dam, further delaying the course of the water, so that except for persons trapped in them, most of the loss of life was among children playing in the streets and women going down to seek them. Terrible as has been the catastrophe, it has left behind almost no one to suffer from the sudden taking away of the bread-winner of a family. The Red Cross Society of Philadelphia sent an agent to care for the orphans but he found only one little girl requiring his care.

As is seen from Dr. Ashcraft's letter, the Austin disaster was peculiar in causing very little surgical and medical work. It either killed outright or left the people unharmed or with minor injuries. Sweeping out a channel bare to the bed rock, it left behind almost no nidus of infection, even decomposing corpses especially in cool weather and with comparatively small aggregation of organic matter, being a very minor source of danger to survivors. Psychic shock and exposure produced some sad mental cases, but these were few in number. Three days after the flood, the local hospital had ten vacant beds and about twenty filled, mostly with patients under treatment before the flood.

